# Culture-based inventory and functional guild analysis of root-invading fungi associated with grassland plants across multiple regions in China

**DOI:** 10.3389/fpls.2026.1869620

**Published:** 2026-07-28

**Authors:** Tong Tong Wang, Bo Yang, Yue Yang Zhang, Hua Qi Liu, Long Bing Zhang, Yan Zhong Li

**Affiliations:** State Key Laboratory of Herbage Improvement and Grassland Agro-Ecosystems, College of Pastoral Agriculture Science and Technology, Lanzhou University; Engineering Research Center of Grassland Industry, Ministry of Education, Lanzhou, China

**Keywords:** culture-based isolation, functional guild, grassland ecosystems, ITS identification, plant-associated fungi, root-invading fungi

## Abstract

Grassland ecosystems harbor diverse plant-associated fungal communities that contribute to ecosystem functioning and plant health. However, large-scale culture-based inventories of root-invading fungi across multiple grassland regions in China remain scarce. In this study, we conducted a multi-region survey of culturable root-invading fungi isolated from surface-sterilized, discolored root and basal stem tissues in contact with the soil. We sampled 33 herbaceous plant species, representing 19 genera and 9 families, across 17 counties in Qinghai, Xinjiang, Inner Mongolia, and Xizang. A total of 273 fungal isolates were preserved and identified using ITS sequence analysis complemented by morphological observations. These isolates were classified into 40 genera and 53 species-level taxa spanning several major fungal lineages. A limited number of genera, including *Fusarium*, *Darksidea*, and *Preussia*, accounted for a substantial proportion of the isolates, whereas numerous low-frequency taxa were also recovered. Fungi were consistently isolated from both root and basal stem tissues, indicating extensive internal colonization of symptomatic tissues across these plant compartments. Significant differences were observed between plant-level and tissue-level isolation rates within both Poaceae and Cyperaceae. Functional guild assignment revealed the presence of endophytic, saprotrophic, and pathogenic taxa, with relative proportions varying among plant groups. Analysis revealed strong fungus-host associations, with Cramer’s *V* of 0.850 indicating a high overall association strength and indicator species analysis identifying eight host-associated taxa. This study provides a culture-based baseline inventory of root-invading fungi associated with natural grassland plants in China and establishes a curated isolate collection for future ecological and functional investigations.

## 1 Introduction

Grassland ecosystems are widely distributed across the globe and account for about 40% of the Earth’s land area. They play key roles in biodiversity conservation, biogeochemical cycling, and the provision of ecosystem services (White et al., 2000). China is one of the five countries with the largest grassland areas worldwide (White et al., 2000). Its grasslands are extensive and highly heterogeneous, comprising 18 distinct types, including alpine meadows, temperate deserts, and alpine grasslands (Liu et al., 2008).

Biodiversity encompasses multiple hierarchical levels, including genetic diversity within species, plant and animal community diversity, and microbial diversity (Bardgett and van der Putten, 2014; Hooper et al., 2005). Increasing evidence demonstrates that soil microorganisms and plant-associated microbes constitute a substantial and functionally critical component of terrestrial biodiversity (Delgado-Baquerizo et al., 2016; Fierer, 2017). Among these microbial groups, fungi are highly diverse and play important ecological roles, participating in processes such as decomposition, nutrient mobilization, and the regulation of plant health (Tedersoo et al., 2014; van der Heijden et al., 2008). Plant-microbe interactions, especially those occurring belowground, play essential roles in carbon sequestration, nutrient cycling, and ecosystem stability in natural systems (Singh et al., 2004). Importantly, biodiversity is closely related to ecosystem functioning, particularly in grassland systems, where higher biodiversity is associated with greater primary productivity, higher stability and resilience, and more efficient nutrient retention and use (Isselstein, 2005). Therefore, understanding the diversity and functional roles of plant-associated fungi is important for explaining their contributions to grassland ecosystem processes.

Fungi that colonize internal plant tissues, hereafter referred to as root-invading fungi, are commonly found in roots and basal stem tissues in contact with the soil. These fungi can act as pathogens or latent endophytes and may affect plant growth, health, and community structure (Fierer, 2017; Tedersoo et al., 2014). Roots, which are in direct contact with the soil, serve as critical interfaces for plant-soil-microbe interactions, providing both entry points and material sources that facilitate fungal colonization (Berg and Smalla, 2009). The colonization process typically involves host recognition, adherence, invasion, colonization, and proliferation (Berg and Smalla, 2009). Depending on environmental conditions and host susceptibility, root-invading fungi may remain asymptomatic or contribute to root rot (Berg and Smalla, 2009). Therefore, characterizing these fungi is important for understanding their roles in plant health and grassland ecosystem functioning.

High-throughput sequencing (HTS) is widely used to characterize fungal communities and has greatly improved our understanding of community composition and diversity across ecosystems (Nilsson et al., 2019a; Peay et al., 2016). However, HTS-based surveys are constrained by incomplete reference databases and cannot replace culture-based approaches for taxonomic validation, functional characterization, and the establishment of reference collections (Hawksworth and Lücking, 2017; Peay et al., 2016). In Chinese grassland ecosystems, recent culture-based studies of plant-associated fungi have mainly focused on specific hosts or habitats, such as alpine medicinal plants (Cui et al., 2015; Hou et al., 2025), desert halophytes (Li et al., 2020), or semiarid grasslands under grazing pressure (Chen et al., 2018). Although these studies have advanced our understanding of host- or habitat-specific fungal assemblages, they are generally limited to single plant taxa in local environments. To our knowledge, large-scale culture-based inventories of root-invading fungi isolated from internal tissues of grassland plants across multiple regions in China remain lacking.

In this study, we conducted a multi-region culture-based survey of root-invading fungi. Fungi were isolated from surface-sterilized roots and basal stems of grassland plants collected from diverse grassland types in China. Using culture-based isolation combined with ITS sequence identification, we aimed to (i) document the species composition of culturable root-invading fungi, (ii) examine host association patterns between fungi and different plant taxa, particularly dominant grassland plant families, and (iii) provide baseline inventory for future studies on fungal diversity and plant-fungus interactions in grassland ecosystems.

## 2 Materials and methods

### 2.1 Study sites and plant sampling

Plant samples were collected from natural grassland ecosystems across multiple regions of China during July and August 2023. Sampling was conducted in 17 survey counties distributed across four major grassland regions: Qinghai Province (Q), Xinjiang Uygur Autonomous Region (X), Inner Mongolia Autonomous Region (I), and Xizang Autonomous Region (T). To minimize spatial autocorrelation, three sampling sites were selected within each county, with a minimum distance of 5,000 m between sites. At each site, three replicate quadrats (1 m × 1 m) were established for plant surveys, and these were spaced at least 100 m apart. Plant surveys were conducted within each quadrat to document the naturally occurring herbaceous species. Sampling strategies differed slightly among regions according to study objectives and local vegetation characteristics. In Q01, X06, and X07, all naturally occurring herbaceous plant species encountered within the quadrats were selected for fungal isolation. In Q05, Q06, Q07, T03, T05, X04, and X05, sampling focused on dominant grassland plant families, specifically Poaceae and Cyperaceae. In Inner Mongolia, one to two dominant plant species at each sampling site were selected for fungal isolation. A minimum of 20 intact individuals of each target plant species were collected per site. Collected plants were carefully excavated to retain complete tissues and were subsequently pressed, dried, and preserved as herbarium voucher specimens. Appropriate measures were taken during sample handling and processing to prevent fungal contamination and mold development prior to isolation. Geographical coordinates (latitude and longitude) and elevation were recorded in the field for each sampling site. Detailed environmental information for each sampling site is summarized in **Table 1**.

**Table 1:** Environmental and sampling information of this study.

Location	Site code	Elevation (m)	Longitude	Latitude	Target plant group(s)
Zhurihe Town, Inner Mongolia	I02	613.90	118°16’E	48°13’N	dominant species
Uliqiy Sumu, Inner Mongolia	I05	1293.66	104°46’E	40°48’N	dominant species
Ulaage, Inner Mongolia	I09	903.60	119°27’E	45°52’N	dominant species
Duolun, Inner Mongolia	I10	1255.70	116°32’E	42°13’N	dominant species
Boluang Tohoi Sumu, Inner Mongolia	I13	630.60	119°11’E	49°29’N	dominant species
Huretu Nuur Sumu, Inner Mongolia	I14	914.30	118°51’E	45°31’N	dominant species
Baiyan Hada Sumu, Inner Mongolia	I15	657.70	119°70’E	49°15’N	dominant species
Menyuan, Qinghai	Q01	3253.33	101°34’E	37°63’N	all herbaceous plants
Wulan, Qinghai	Q05	3193.56	98°43’E	36°80’N	Poaceae and Cyperaceae
Dulan, Qinghai	Q06	3059.90	98°80’E	36°20’N	Poaceae and Cyperaceae
Tianjun, Qinghai	Q07	3483.62	98°41’E	37°31’N	Poaceae and Cyperaceae
Amdo, Xizang	T03	4491.87	92°30’E	31°28’N	Poaceae and Cyperaceae
Shanzha, Xizang	T05	4590.00	89°00′E	31°24′N	Poaceae and Cyperaceae
Zhaosu, Xinjiang	X04	1595.03	81°10’E	42°59’N	Poaceae and Cyperaceae
Tacheng City, Xinjiang	X05	407.87	83°10’E	46°35’N	Poaceae and Cyperaceae
Altay City, Xinjiang	X06	1274.70	87°43’E	48°00’N	all herbaceous plants
Fuyun, Xinjiang	X07	1191.50	89°52’E	47°13’N	all herbaceous plants

Target plant group(s) indicate the plant group(s) selected for fungal isolation at each survey county.

### 2.2 Plant identification

To identify plants, a comprehensive approach that included both molecular evidence and morphological examination was employed. The genomic DNA was extracted from dry plant tissues using the HP Plant DNA Kit D2485 (Omega Bio-tek, Inc., GA, USA). The internal transcribed spacer (ITS) region of ribosomal DNA was amplified using universal primers ITS1 (5’-TCCGTAGGTGAACCTGCGG-3’) and ITS4 (5’-TCCTCCGCTTATTGATATGC-3’) (White et al., 1990). PCR amplifications were performed in a 25 μL reaction volume containing 1 μL template DNA, 1 μL of each primer, 12.5 μL 2×Taq PCR Master Mix, and 9.5 μL ddH_2_O. Thermal cycling conditions consisted of an initial denaturation at 94°C for 5 min; followed by 35 cycles of 94°C for 30 s, 55°C for 30 s, and 72°C for 30 s; with a final extension at 72°C for 10 min. PCR products were sequenced by Tsingke Biotechnology Co., Ltd. (Beijing, China). For specimens with ITS sequences that were successfully acquired, these sequences were matched against the NCBI database using BLASTn to propose initial taxonomic hypotheses. Final identification for all specimens was confirmed by Prof. Yuhu Wu (Northwest Plateau Institute of Biology) through detailed morphological examination and comparison with validated taxonomic descriptions in Flora Reipublicae Popularis Sinicae (https://www.iplant.cn/frps) and the National Specimen Information Infrastructure (http://www.nsii.org.cn/2017/home.php). Geographic distribution and habitat information were also considered as supporting evidence. For specimens without successful DNA extraction or PCR amplification, identification was based solely on morphological examination. One Poaceae specimen could not be confidently resolved to the species level even after expert consultation and was therefore treated at the family level in subsequent analyses.

### 2.3 Fungal isolation and cultivation

Fungi were isolated using a tissue-segment isolation method. This study aimed to evaluate whether fungi isolated from natural grassland plants were potential root rot-associated pathogens. Therefore, isolation was conducted from both discolored root tissues and basal stem tissues in direct contact with soil. Although basal stems did not consistently exhibit visible discoloration, they were included because repeated isolation of identical fungal taxa from both roots and basal stems within the same plant would increase the likelihood that the fungus was actively colonizing internal plant tissues. Freshly collected plant samples were processed shortly after collection. Plant tissues (basal stems and roots) were cut into 2 mm^3^ segments and surface-sterilized (75% ethanol, 30 s; 1% NaClO, 2 min) to remove epiphytic microorganisms, rinsed three times with sterile distilled water, and then dried on sterile filter paper. Five individual plants per site were selected for isolation. Three plates were prepared per tissue type, and five tissue segments were placed on each plate. Surface-sterilized tissue segments were placed on potato dextrose agar (PDA; Beijing Land Bridge Technology Co., Ltd., Beijing, China) supplemented with antibiotics (200 mg/L penicillin and 200 mg/L streptomycin). Plates were examined regularly for fungal growth. To improve the reliability of fungal species records and to minimize the inclusion of sporadic or contaminant fungi, only fungal taxa that were isolated at least three times were selected for further purification and identification. Fungal isolates with lower isolation frequencies were recorded during isolation but were not subjected to purification or DNA sequencing. During this process, plant-level isolation rate (Equation 1), tissue-level isolation rate (Equation 2), root isolation rate (Equation 3), and basal stem isolation rate (Equation 4) were recorded. The purified isolates were stored at 4°C and -80°C in the Strain Preservation Library of the College of Pastoral Agriculture Science and Technology of Lanzhou University.

(1) $$plant-levelisolationrate(\%)=\frac{numberofplantsisolatedthetargetfungi}{totalnumberofplants}\times 100$$

(2) $$tissue–levelisolationrate(\%)=\frac{numberoftissuesisolatedthetargetfungi}{totalnumberoftissues}\times 100$$

(3) $$rootisolationrate(\%)=\frac{numberofroottissuesisolatedthetargetfungi}{totalnumberofroottissues}\times 100$$

(4) $$basalstemisolationrate(\%)=\frac{numberofbasalstemtissuesisolatedthetargetfungi}{totalnumberofbasalstemtissues}\times 100$$

### 2.4 DNA extraction, PCR amplification, and sequencing

Genomic DNA was extracted from fresh fungal mycelia grown on PDA using an E.Z.N.A.^®^ High Performance Fungal DNA Kit (Omega Bio-tek, Inc., GA, USA). The ITS region was amplified using primers ITS1 and ITS4 under the same PCR conditions described in Section 2.2. Sequencing procedures were also conducted as described above. Obtained ITS sequences were compared with reference sequences in the NCBI GenBank database using BLASTn. Taxonomic identity was assigned according to sequence similarity and query coverage thresholds commonly applied in fungal molecular taxonomy. Species-level identification was accepted when sequence similarity was ≥99% and query coverage ≥95%, whereas isolates with similarity of 97-99% were assigned to genus level (Nilsson et al., 2019b; Schoch et al., 2012). When ITS sequences showed high similarity to multiple closely related species and could not be confidently resolved to a single species, morphological characteristics of the fungi (sporulating structures, conidia, etc.) were examined to support taxonomic assignment. Morphological observations were used as complementary evidence rather than as a primary identification criterion. Functional guilds of the identified fungi (e.g., plant pathogens, endophytes, or saprotrophs) were assigned based on Fungal Genus (https://fungalgenera.org/) and published literature, and their relative proportions (Equation 5) were recorded. These functional guilds were further corroborated by pathogenicity evidence from previous inoculation experiments conducted on selected isolates from the same collection, including both published and unpublished data.

(5) $$relativeproportions(\%)=\frac{numberofisolatesassignedtoafunctionalguilds}{totalnumberofisolatesfromthatplantgroup}\times 100$$

### 2.5 Data analysis

As isolation rate data did not meet the assumption of normality (Shapiro-Wilk test), nonparametric methods were applied. Differences among plant groups and tissue types were evaluated using the Kruskal-Wallis test followed by Dunn’s *post-hoc* test with Bonferroni correction for pairwise comparisons in R v.4.4.3.

Associations between fungal species and host plants were assessed using contingency table analysis. Overall association strength was quantified using Cramer’s *V* coefficient, and significant fungus-host combinations were identified using adjusted residual analysis (adjusted residual ≥ |2.0|) in SPSS v.21.

Host preference of fungal species was further evaluated using Indicator Species Analysis implemented with the strassoc function in the R v.4.4.3 package indicspecies (De Cáceres and Legendre, 2009). Indicator values (IndVal) ≥ 0.70 were considered to indicate strong host associations (Dufrêne and Legendre, 1997). Data visualization was performed using GraphPad Prism v.8.0.2 (GraphPad Software, CA, USA).

## 3 Results

### 3.1 Isolation patterns of culturable fungi across plant groups

A total of 33 herbaceous plant species representing 19 genera and 9 families were collected from natural grasslands across the surveyed regions. The sampled plant assemblages were dominated by Poaceae and Cyperaceae; Cyperaceae was consistently present in Xizang, whereas Poaceae was consistently present at the other survey sites. Culturable fungi were isolated from discolored root tissues and basal stem tissues of plants belonging to Poaceae, Cyperaceae, and other herbaceous families. Across all sampled plants, fungi were isolated from multiple plant individuals and tissue segments, indicating widespread colonization of grassland plants by culturable fungi.

**Figure 1:**
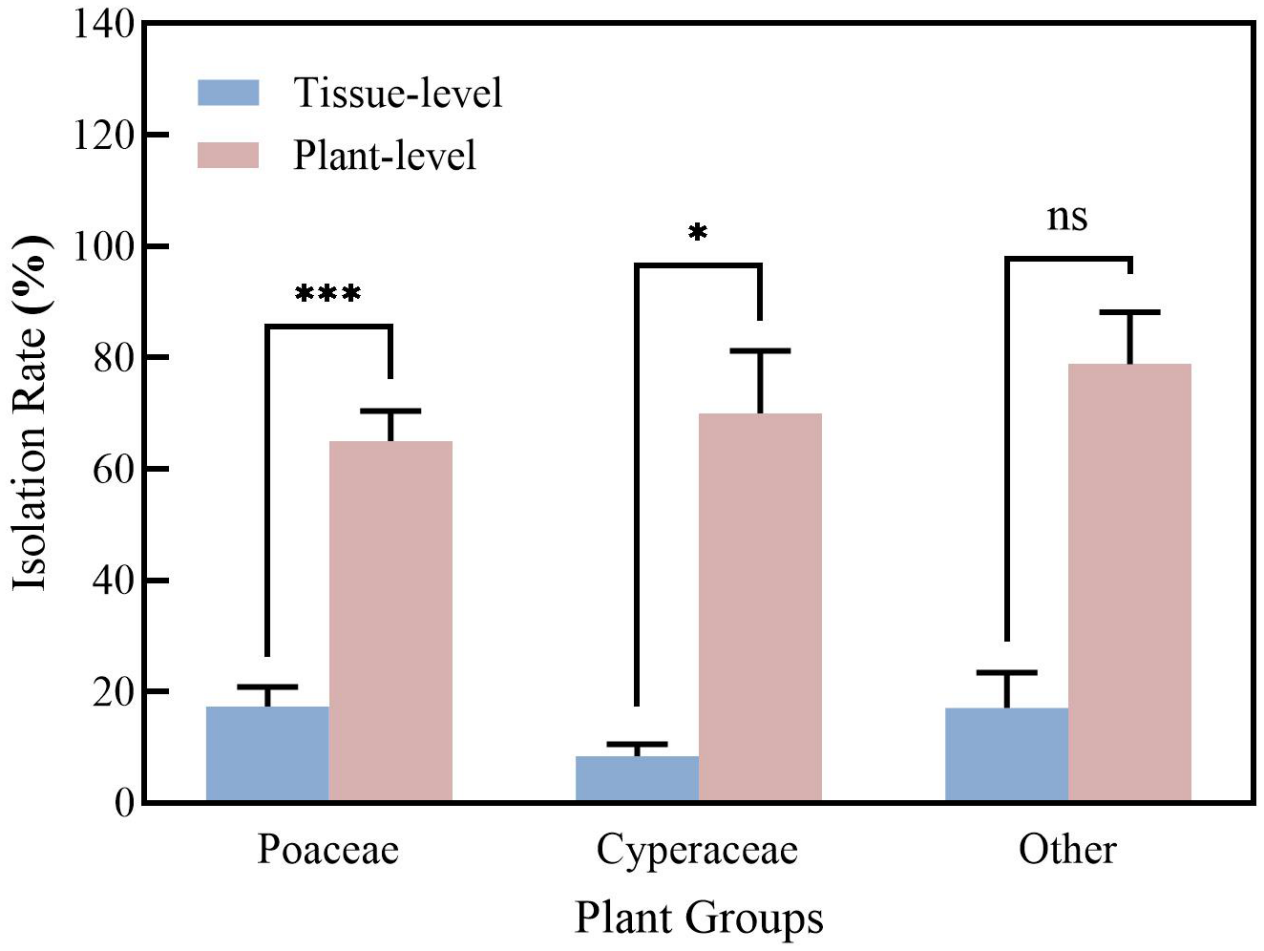
Tissue- and plant-level isolation rates of culturable fungi associated with different grassland plant groups. Bars represent mean ± SEM. *means *P* < 0.05; *** means *P* < 0.001; ns means not significant. No significant differences were observed among plant groups at either the plant or tissue level (Kruskal-Wallis test with Dunn’s *post-hoc* and Bonferroni correction).

The Kruskal-Wallis test revealed significant differences among the six plant group–isolation level categories (*χ²* = 32.128, df = 5, *P* < 0.001) (**Figure 1**). Pairwise comparisons with Bonferroni correction showed that plant-level isolation rates were significantly higher than tissue-level isolation rates within each group (Cyperaceae: corrected *P* < 0.05; Poaceae: corrected *P* < 0.001). However, no significant differences were detected among Cyperaceae, Poaceae, and other plant groups at either the plant or tissue level (corrected *P* > 0.05).

### 3.2 Taxonomic composition of culturable fungi

**Figure 2:**
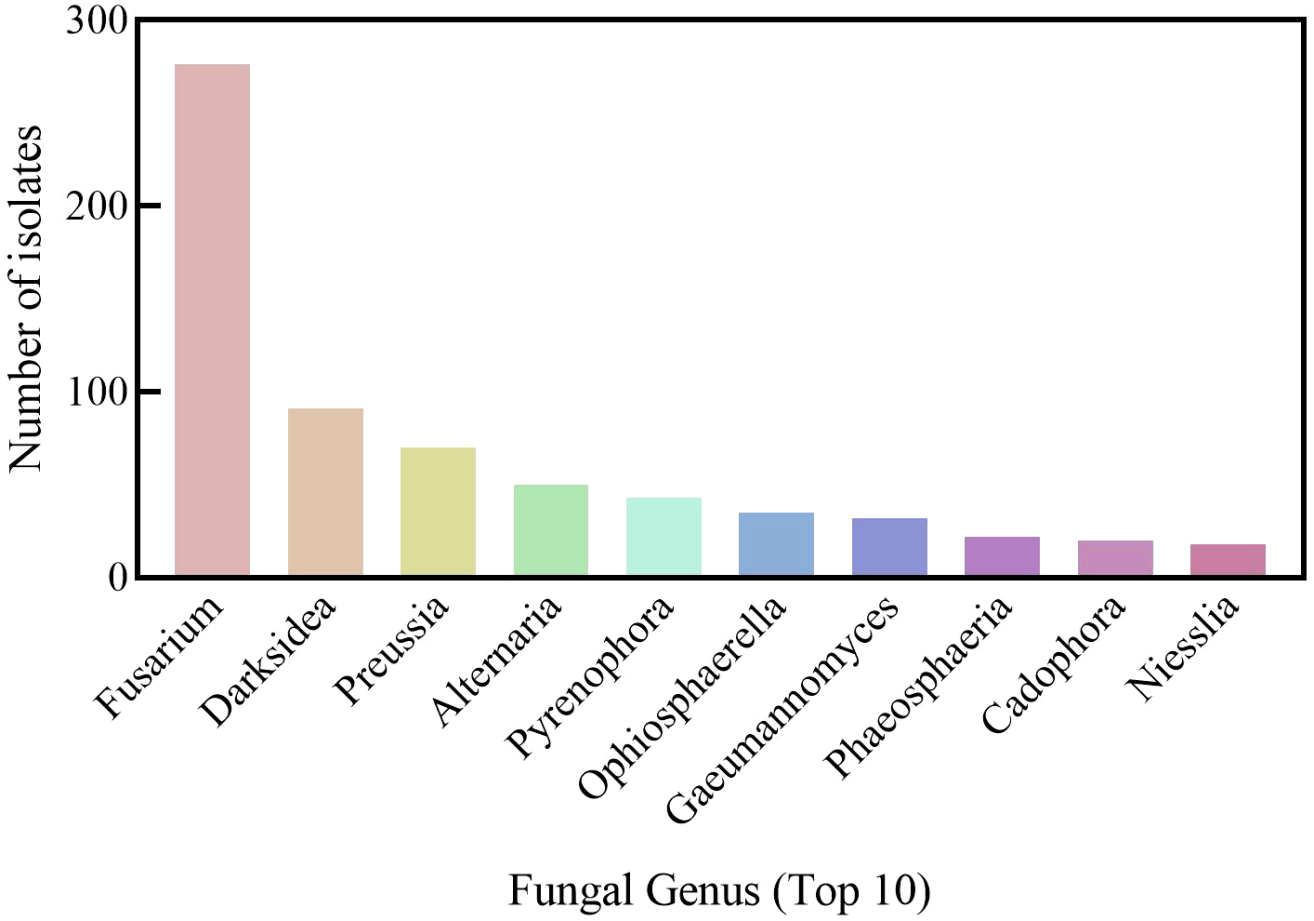
Top 10 taxonomic composition of culturable fungi isolated from grassland plants.

A total of 273 fungal isolates were preserved for molecular identification. These isolates were assigned to 40 genera and 53 species-level taxa spanning several major fungal lineages. Detailed taxonomic information, including isolate codes, host plants, isolation tissues, ITS accession numbers, and sequence similarity values, is provided in **Supplementary Table 1**. A few genera accounted for a relatively large proportion of the total isolates, including *Fusarium*, *Darksidea*, and *Preussia*, whereas the remaining genera were represented by fewer isolates (**Figure 2**). Several genera, including *Fusarium* and *Darksidea*, were recovered from multiple host species. Several rare fungal taxa were also detected, including *Seimatosporium pistaciae*, *Alpinaria rhododendri*, and *Austropleospora keteleeriae*.

### 3.3 Tissue-specific isolation patterns in Poaceae and Cyperaceae

**Figure 3:**
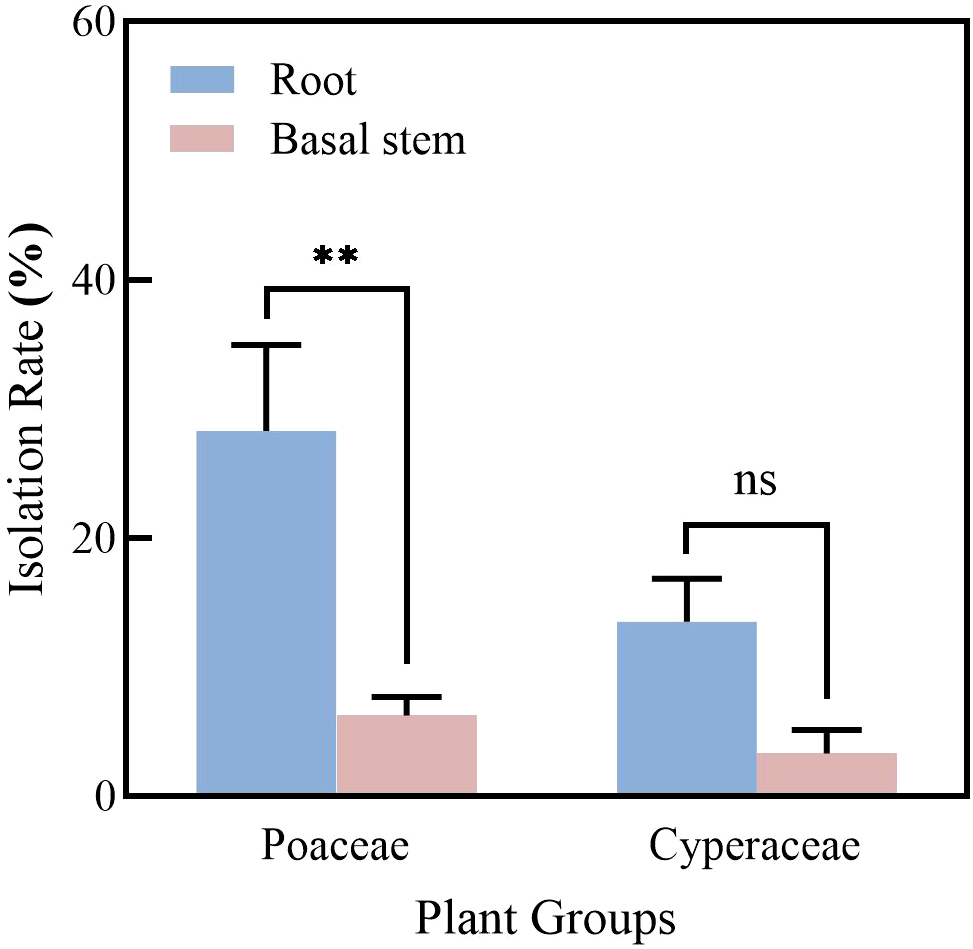
Isolation rates of culturable fungi from different plant tissues. Bars represent mean ± SEM. ** means *P* < 0.01; ns means not significant (Kruskal-Wallis test with Dunn’s *post-hoc* and Bonferroni correction).

Culturable fungi were consistently isolated from root and basal stem tissues in both Poaceae and Cyperaceae, indicating that multiple plant compartments serve as niches for fungal colonization. The Kruskal-Wallis test revealed significant differences among the four plant group – tissue type combinations (*χ²* = 17.111, df = 3, *P* < 0.001). Pairwise comparisons with Bonferroni correction showed that fungal isolation rates from roots were significantly higher than those from basal stems in Poaceae (corrected *P* < 0.01; **Figure 3**). In Cyperaceae, the difference between root and basal stem isolation rates did not reach statistical significance after correction, although the isolation rate remained numerically higher in roots. No significant differences were found between Poaceae and Cyperaceae at either the root or basal stem level (corrected *P* > 0.05).

### 3.4 Functional guilds of culturable fungi associated with grassland plants

**Figure 4:**
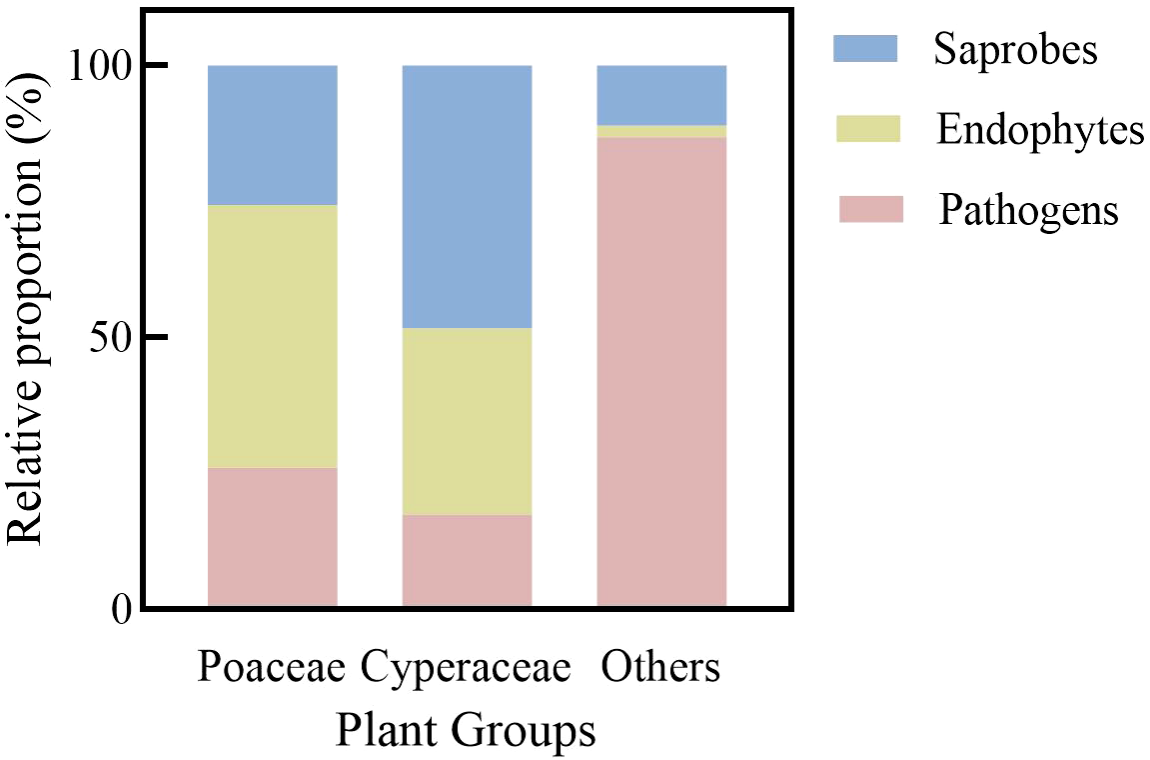
Functional guilds composition of culturable fungi associated with different plant groups.

Fungal isolates were assigned to different functional guilds, including endophytes, saprobes, and pathogens (**Supplementary Table 2**). The relative proportions of these functional guilds varied among plant groups (**Figure 4**). Poaceae harbored a relatively higher proportion of endophytes, whereas Cyperaceae and other herbaceous plants showed increased representation of saprotrophic and pathogenic taxa.

To complement taxonomy-based functional assignments, available pathogenicity evidence was compiled for selected isolates originating from the present collection. Three species (*Alternaria chlamydosporigena*, *Fusarium acuminatum*, and *Rhypophila decipiens*) had previously fulfilled Koch’s postulates on their host plants and were confirmed as pathogenic in earlier studies by our group (Wang and Li, 2025a, 2025b, 2026). In contrast, inoculation assays conducted on four additional isolates (*Niesslia aemula* inoculated on *Leymus flexus*, *Seimatosporium pistaciae* inoculated on *Potentilla pensylvanica*, *Cadophora luteo-olivacea* inoculated on *Plantago asiatica*, and *Leptosphaeria* sp. inoculated on *Elymus nutans*) did not induce disease symptoms under experimental conditions. These observations indicate that isolates recovered from internal plant tissues may encompass both pathogenic and non-pathogenic ecological strategies.

### 3.5 Fungus-host associations

Global association analysis revealed a strong association (Cramer’s *V* = 0.850) between fungal taxa and host plant species, suggesting that fungal occurrence patterns varied among host species.

Eight fungal taxa showed high indicator values (IndVal ≥ 0.70) (**Table 2**). Among these, *Austropleospora keteleeriae* exhibited the strongest host association with *Artemisia japonica* (IndVal = 0.952), *Rhypophila decipiens* with *Deyeuxia pyramidalis* (IndVal = 0.945), and *Seimatosporium pistaciae* for *Potentilla pensylvanica* (IndVal = 0.919). Within the genus *Fusarium*, multiple species exhibited distinct host preferences: *F. acuminatum* showed extremely strong indication for *A. schanginianus* (IndVal = 0.950); *F. incarnatum-equiseti* species complex (IndVal = 0.887) and *F. tricinctum* species complex (IndVal = 0.773) both showed strong indication for *Peganum harmala*. Additionally, *Preussia terricola* was strongly associated with *Achnatherum psilantherum* (IndVal = 0.845), and *Phoma conidiogena* with *Peganum harmala* (IndVal = 0.725). Adjusted residual analysis identified significant enrichment (adjusted residual ≥ |2.0|) for these fungus-host combinations (**Supplementary Table 3**).

**Table 2:** Strong indicator fungus-host associations (IndVal ≥ 0.70) based on IndVal analysis.

Fungal species	Indicator host	IndVal
*Austropleospora keteleeriae*	*Artemisia japonica*	0.952
*Fusarium acuminatum*	*Astragalus schanginianus*	0.950
*Rhypophila decipiens*	*Deyeuxia pyramidalis*	0.945
*Seimatosporium pistaciae*	*Potentilla pensylvanica*	0.919
*Fusarium incarnatum-equiseti species complex*	*Peganum harmala*	0.887
*Preussia terricola*	*Achnatherum psilantherum*	0.845
*Fusarium tricinctum species complex*	*Peganum harmala*	0.773
*Phoma conidiogena*	*Peganum harmala*	0.725

## 4 Discussion

### 4.1 Culture-based surveys reveal widespread root colonization in grassland plants

This multi-region survey indicates that culturable root-invading fungi are widely distributed across natural grassland ecosystems in China. Fungi were repeatedly isolated from both root and basal stem tissues of diverse herbaceous hosts, indicating that internal colonization of belowground plant tissues is common in grassland systems. Consistent with this observation, statistical comparisons showed that isolation rates differed primarily between plant-level and tissue-level measurements rather than among host groups, suggesting that differences in fungal recovery were more strongly associated with sampling scale than with broad plant taxonomic categories. Compared with HTS studies, which often reveal high hidden diversity, culture-based methods provide taxonomically confirmed isolates. These isolates can be preserved, experimentally tested, and used for functional studies (Hawksworth and Lücking, 2017; Peay et al., 2016). Although culture-based methods may underestimate total fungal richness, they are important for linking taxonomy with phenotype and ecological function (Hawksworth and Lücking, 2017; Hyde et al., 2019; Peay et al., 2016). Therefore, this study provides a reference collection and baseline inventory for future ecological and experimental research.

### 4.2 Taxonomic structure dominated by a limited number of recurrent genera

A small number of genera, including *Fusarium*, *Darksidea*, and *Preussia*, accounted for most isolates. Such a pattern is common in culture-based fungal studies (Collado et al., 1999), where fast-growing or adaptable taxa are more easily recovered under laboratory conditions (Hyde et al., 2019). The repeated occurrence of these genera across different hosts and regions suggests broad ecological adaptability and possible host generalist traits. *Darksidea*, in particular, has been widely reported as a root endophyte in grassland and dryland ecosystems and is often linked to stress tolerance and persistence in soil (Knapp et al., 2015). In addition, several rare taxa were detected. Although rare, their repeated isolation under sterile conditions supports a true association with plant tissues rather than contamination. These taxa may represent underexplored components of grassland fungal diversity (Jousset et al., 2017; Lynch and Neufeld, 2015).

### 4.3 Tissue compartment as an ecological niche

Fungal recovery from both roots and basal stems indicates that multiple plant compartments can serve as colonization niches. Statistical comparisons further supported this pattern, showing significantly higher isolation rates in Poaceae roots than in basal stems, whereas Cyperaceae exhibited less pronounced tissue differentiation. This result suggests that tissue compartment may influence fungal recovery differently across host groups. Rather than reflecting strict differences, these patterns suggest that tissue compartment is an important factor shaping fungal distribution. Roots, as the main interface with soil, are continuously exposed to soilborne propagules (Berg and Smalla, 2009). In contrast, basal stems may act as transitional zones between belowground and aboveground tissues (Compant et al., 2005). These results support the view that internal plant tissues form heterogeneous microhabitats that influence fungal colonization and persistence.

### 4.4 Functional guilds and pathogenic potential

Functional guild classification based on taxonomy does not always reflect actual ecological roles. Many fungi show flexible lifestyles and can switch among endophytic, saprotrophic, or pathogenic modes depending on environmental conditions (Porras-Alfaro and Bayman, 2011; Schulz and Boyle, 2005). Notably, three isolated species had previously fulfilled Koch’s postulates on their host plants and were confirmed as pathogenic (Wang and Li, 2025a, 2025b, 2026), whereas four taxa were demonstrated to be non-pathogenic under controlled inoculation assays. The presence of both pathogenic and non-pathogenic fungi in internal tissues indicates diverse ecological roles among root-invading fungal communities. This coexistence highlights the complexity of root-invading communities in grassland plants. As in agricultural systems, disease outcomes in natural grasslands likely depend on interactions among environmental conditions, host susceptibility, pathogen virulence, and the plant microbiome (Berg and Raaijmakers, 2018; Burdon and Thrall, 2008). These interacting factors may determine whether fungi remain asymptomatic or become pathogenic when hosts are under stress.

### 4.5 Methodological considerations and limitations

Several limitations should be noted. First, sampling strategies differed among regions, particularly in host selection. This may affect the observed taxonomic composition. Therefore, the dataset should be considered a large-scale inventory rather than a strictly comparable regional analysis. Second, culture-based methods select for fungi that can grow under laboratory conditions. As a result, obligate biotrophs and slow-growing taxa may be underrepresented (Hibbett et al., 2016; Lindahl et al., 2013). Despite these limitations, this study provides a well-documented isolate collection linked to host identity and geographic origin. This resource offers a strong foundation for future functional, genomic, and pathogenicity research.

### 4.6 Host preference of culturable root-invading fungi

The strong fungus-host associations revealed by Cramer’s *V* and the elevated indicator values observed in IndVal analysis suggest that host identity may contribute to variation in culturable fungal occurrence patterns within grassland communities. Several fungal taxa showed pronounced host-associated occurrence patterns. In particular, multiple *Fusarium* taxa displayed relatively high indicator values for different host plants, suggesting that closely related species may differ in their host associations in natural ecosystems. Although the present data do not directly demonstrate host specialization, these observations are consistent with the idea that host preference may influence the ecological distribution of certain fungal taxa. Host-associated differentiation has been extensively documented in some *Fusarium* lineages, particularly through the concept of formae speciales in *F. oxysporum*, where pathogenic populations exhibit distinct host ranges (Edel-Hermann and Lecomte, 2019). While the present study does not provide experimental evidence for comparable specialization, the observed host-associated patterns indicate that similar ecological processes deserve further investigation in culturable fungi from natural grassland systems.

These findings contribute to the current understanding of belowground culturable host-associated fungal diversity in grassland ecosystems, which remains less explored than aboveground communities (Bardgett and van der Putten, 2014; Delgado-Baquerizo et al., 2016). Future studies integrating culture-based methods, HTS, functional characterization, and environmental data will further clarify the ecological roles of these fungi and their contributions to grassland ecosystem stability.

## 5 Conclusions

This study provides a large-scale culture-based inventory and functional guild analysis of root-invading fungi associated with natural grassland plants across multiple regions of China. A total of 273 isolates representing 40 genera and 53 species-level taxa were recovered from roots and basal stems of 33 herbaceous plant species. A limited number of genera accounted for a substantial proportion of isolates, whereas numerous low-frequency taxa were also detected. Fungi were consistently recovered from both root and basal stem tissues of the sampled plant species, indicating widespread internal colonization of symptomatic tissues in multiple plant compartments. Functional guild assignment revealed the coexistence of endophytic, saprotrophic, and pathogenic taxa within grassland plants, underscoring the ecological complexity of these root-invading fungal communities. Statistical analysis revealed strong fungus-host associations and host-related variation in fungal occurrence patterns. Together, these results provide a curated reference collection and a baseline inventory of culturable root-invading fungi for future ecological and functional studies of plant-associated fungi in grassland ecosystems.

## Data Availability

The datasets presented in this study can be found in online repositories. The names of the repository/repositories and accession number(s) can be found in the article/**Supplementary Material**.
