# Supplementary material for: Culture-based inventory and functional guild analysis of root-invading fungi associated with grassland plants across multiple regions in China
Source: Front Plant Sci. 2026 Jul 28;17:1869620. doi: 10.3389/fpls.2026.1869620 (PMC13457472; doi:10.3389/fpls.2026.1869620)
Supplement: Supplementary file 1 [file Table1.docx]

Supplementary Table 1. List of preserved and identified culturable fungi isolated from grassland plants

| Host | | Strain information | | | | | Numbers | Province | Isolation source |
| --- | --- | --- | --- | --- | --- | --- | --- | --- | --- |
| Family | Species | Strain | ITS number | Most probable taxonomic matches | | percent identity (%) |  |  |  |
| Poaceae | *Achnatherum psilantherum* | LYZ1283-1286;  LYZ1552-1553 | PX979195-979198; PX979317-979318 | *Preussia terricola* | MW548076 | 99.38 | 51 | Q06 | R |
| Poaceae | *Achnatherum psilantherum* | LYZ1287-1290 | PX979199-979202 | *Fusarium incarnatum-equiseti* species complex | KT192255 | 100 | 15 | Q06 | R; BS |
| Poaceae | *Achnatherum psilantherum* | LYZ1544-1545 | PX979309-979310 | *Slopeiomyces cylindrosporus* | MK102668 | 100 | 7 | Q06 | R |
| Poaceae | *Achnatherum psilantherum* | LYZ1546-1551 | PX979311-979316 | *Ophiosphaerella* sp. | KY987527 | 97.69 | 20 | Q06 | R |
| Poaceae | *Achnatherum psilantherum* | LYZ1619-1620 | PX979382-979383 | *Fusarium tricinctum* species complex | OP223065 | 100 | 7 | Q06 | R; BS |
| Poaceae | *Achnatherum regelianum* | LYZ1225-1228 | PX979137-979140 | *Darksidea* sp. | ON074787 | 98.20 | 4 | I05 | R |
| Poaceae | *Achnatherum regelianum* | LYZ1229-1232 | PX979141-979144 | *Preussia terricola* | MW548076 | 99.80 | 6 | I05 | R |
| Poaceae | *Achnatherum regelianum* | LYZ1233-1236 | PX979145-979148 | *Fusarium incarnatum-equiseti* species complex | OM956029 | 100 | 15 | I05 | R |
| Poaceae | *Achnatherum regelianum* | LYZ1237-1240 | PX979149-979152 | *Fusarium nygamai* | PX788640 | 100 | 24 | I05 | R |
| Poaceae | *Achnatherum regelianum* | LYZ1521-1524 | PX979286-979289 | *Poaceascoma* sp. | MT683271 | 95.84 | 15 | I05 | R |
| Poaceae | *Achnatherum splendens* | LYZ1241-1244 | PX979153-979156 | *Darksidea alpha* | JN859359 | 100 | 15 | Q05 | R |
| Poaceae | *Achnatherum splendens* | LYZ1245-1248 | PX979157-979160 | *Microdochium bolleyi* | MT446085 | 100 | 8 | Q05 | R; BS |
| Poaceae | *Achnatherum splendens* | LYZ1559-1562 | PX979324-979327 | *Knufia tsunedae* | OR105795 | 100 | 15 | Q05 | R; BS |
| Poaceae | *Achnatherum splendens* | LYZ1563-1565 | PX979328-979330 | *Ophiosphaerella* sp. | KT270209 | 98.11 | 15 | Q05 | R |
| Poaceae | *Achnatherum splendens* | LYZ1566-1567 | PX979331-979332 | *Phaeosphaeria* sp. | OM237127 | 99.63 | 7 | Q05 | R; BS |
| Poaceae | *Achnatherum splendens* | LYZ1354-1357 | PX979258-979261 | *Crinipellis scabella* | PQ653119 | 99.85 | 4 | Q06 | R |
| Poaceae | *Achnatherum splendens* | LYZ1585-1586 | PX979350-979351 | *Darksidea alpha* | JN859359 | 99.64 | 20 | Q06 | R; BS |
| Poaceae | *Achnatherum splendens* | LYZ1499-1506 | PX979267-979274 | *Darksidea* sp. | OK037167 | 98.58 | 31 | X05 | R |
| Poaceae | *Achnatherum splendens* | LYZ1507-1509 | PX979275-979277 | *Fusarium concolor* | MW995751 | 99.81 | 4 | X05 | R |
| Poaceae | *Agropyron cristatum* | LYZ1327-1330 | PX979231-979234 | *Gaeumannomyces hyphopodioides* | KX306510 | 99.18 | 11 | I10 | R |
| Poaceae | *Agropyron cristatum* | LYZ1331-1334 | PX979235-979238 | *Periconia macrospinosa* | MT683272 | 100 | 7 | I10 | R |
| Poaceae | *Agropyron cristatum* | LYZ1496-1498 | PX979264-979266 | **Pleosporales** | MW995686 | 99.61 | 4 | I10 | R |
| Poaceae | *Agropyron cristatum* | LYZ1526 | PX979291 | *Leratiomyces laetissimus* | HF912362 | 99.53 | 3 | I10 | R; BS |
| Poaceae | *Agropyron cristatum* | LYZ1527-1528 | PX979292-979293 | *Marasmius* sp. | EF187911 | 98.91 | 5 | I10 | BS |
| Poaceae | *Agropyron cristatum* | LYZ1529 | PX979294 | *Alternaria longissima* | MN517853 | 100 | 3 | I10 | R |
| Poaceae | *Bromus inermis* | LYZ1303-1306 | PX979207-979210 | **Xylariales** | PP336516 | 98.38 | 5 | X04 | BS |
| Poaceae | *Bromus inermis* | LYZ1538-1539 | PX979303-979304 | *Darksidea* sp. | OK037167 | 99.03 | 3 | X04 | R |
| Poaceae | *Bromus inermis* | LYZ1540-1541 | PX979305-979306 | *Phaeosphaeria pontiformis* | PP171736 | 100 | 3 | X04 | BS |
| Poaceae | *Bromus inermis* | LYZ1542-1543 | PX979307-979308 | *Slopeiomyces cylindrosporus* | KY430509 | 100 | 3 | X04 | R |
| Poaceae | *Bromus inermis* | LYZ1617-1618 | PX979380-979381 | *Darksidea alpha* | KP183967 | 100 | 7 | X04 | R |
| Poaceae | *Deyeuxia pyramidalis* | LYZ1510-1512 | PV933831-933833 | *Rhypophila decipiens* | PV933831 | 100 | 4 | Q01 | R |
| Poaceae | *Elymus nutans* | LYZ1202-1204 | PX979121-979123 | *Neoascochyta europaea* | PP639659 | 100 | 3 | Q01 | R |
| Poaceae | *Elymus nutans* | LYZ1589-1590 | PX979354-979355 | *Cercophora* sp. | ON193749 | 100 | 6 | Q07 | R |
| Poaceae | *Elymus nutans* | LYZ1591-1592 | PX979356-979357 | *Leptosphaeria* sp. | KT268775 | 99.34 | 7 | Q07 | R; BS |
| Poaceae | *Elymus nutans* | LYZ1597-1598 | PX979360-979361 | *Darksidea alpha* | JN859359 | 99.81 | 8 | Q07 | R |
| Poaceae | *Leymus chinensis* | LYZ1221-1224 | PX979133-979136 | *Microdochium trichocladiopsis* | KP858998 | 100 | 6 | I09 | R |
| Poaceae | *Leymus chinensis* | LYZ1645-1648 | PX979408-979411 | *Phaeosphaeria pontiformis* | MW188650 | 99.82 | 6 | I01 | R |
| Poaceae | *Leymus chinensis* | LYZ1649-1652 | PX979412-979415 | *Albotricha* sp. | JN995639 | 98.55 | 13 | I01 | BS |
| Poaceae | *Leymus chinensis* | LYZ1653-1656 | PX979416-979419 | *Lachnum* sp. | MK808062 | 99.20 | 4 | I01 | R |
| Poaceae | *Leymus flexus* | LYZ1249-1252 | PX979161-979164 | *Preussia terricola* | OP710153 | 99.05 | 13 | Q05 | BS |
| Poaceae | *Leymus flexus* | LYZ1319-1322 | PX979223-979226 | *Niesslia aemula* | MG826957 | 99.32 | 18 | Q05 | R; BS |
| Poaceae | *Leymus secalinus* | LYZ1554-1555 | PX979319-979320 | *Fusarium oxysporum* species complex | PQ302565 | 100 | 11 | I05 | R |
| Poaceae | *Leymus secalinus* | LYZ1556-1558 | PX979321-979323 | *Leratiomyces laetissimus* | MH043619 | 99.69 | 8 | I05 | BS |
| Poaceae | *Poa albertii* | LYZ1295-1302 | PV926699-926706 | *Alternaria chlamydosporigena* | MH863797 | 99.65 | 10 | Q05 | R |
| Poaceae | *Puccinellia roshevitsiana* | LYZ1576-1578 | PX979341-979343 | **Sordariales** | MT679674 | 96.17 | 3 | T05 | R |
| Poaceae | *Stipa penicillata* | LYZ1343-1346 | PX979247-979259 | *Colletotrichum spaethianum* | PV423075 | 100 | 6 | Q01 | BS |
| Poaceae | *Stipa penicillata* | LYZ1604-1605 | PX979367-979368 | *Phialophora mustea* | JX145398 | 100 | 3 | Q01 | R |
| Poaceae | *Stipa penicillata* | LYZ1606-1607 | PX979369-979370 | **Helotiales** | DQ350130 | 99.06 | 3 | Q01 | R |
| Poaceae | *Stipa purpurea* | LYZ1212-1215 | PX979124-979127 | *Limonomyces* sp. | OP039324 | 95.79 | 8 | Q01 | BS |
| Poaceae | *Stipa purpurea* | LYZ1220 | PX979132 | *Septoriella* sp. | NR_164461 | 98.93 | 6 | Q01 | R; BS |
| Poaceae | unconfirmed | LYZ1613-1616 | PX979376-979379 | **Pleosporales** | GQ924022 | 95.15 | 13 | X06 | R |
| Cyperaceae | *Carex alatauensis* | LYZ1315-1318 | PX979219-979222 | *Coniochaeta* sp. | PQ632316 | 100 | 15 | T03 | R; BS |
| Cyperaceae | *Carex alatauensis* | LYZ1587-1588 | PX979352-979353 | **Sordariales** | MT242295 | 100 | 6 | T03 | R |
| Cyperaceae | *Carex haematostoma* | LYZ1530-1532 | PX979295-979297 | *Microscypha* sp. | PQ286970 | 99.62 | 3 | T03 | R |
| Cyperaceae | *Carex haematostoma* | LYZ1533-1535 | PX979298-979300 | **Chaetothyriales** | GQ923989 | 100 | 3 | T03 | R |
| Cyperaceae | *Carex kokanica* | LYZ1581-1582 | PX979346-979347 | *Cistella* sp. | OR733540 | 98.44 | 8 | T03 | R |
| Cyperaceae | *Carex kokanica* | LYZ1583-1584 | PX979348-979349 | *Darksidea* sp. | MK808199 | 95.25 | 3 | T03 | R |
| Cyperaceae | *Carex turkestanica* | LYZ1272-1274 | PX979184-979186 | *Paraphaeosphaeria* sp. | NR_155698 | 98.04 | 6 | X04 | R; BS |
| Cyperaceae | *Carex turkestanica* | LYZ1572-1575 | PX979337-979340 | *Gaeumannomyces* sp. | PV806599 | 95.17 | 21 | X04 | R; BS |
| Cyperaceae | *Carex turkestanica* | LYZ1579-1580 | PX979344-979345 | *Heteroconium* sp. | MZ380069 | 99.05 | 11 | X04 | R; BS |
| Asteraceae | *Achillea millefolium* | LYZ1311-1314 | PX979215-979218 | *Plenodomus tracheiphilus* | MK460905 | 100 | 9 | X07 | BS |
| Asteraceae | *Artemisia japonica* | LYZ1347-1350 | PX979251-979254 | *Austropleospora keteleeriae* | OR760527 | 99.65 | 10 | X06 | R; BS |
| Asteraceae | *Artemisia japonica* | LYZ1609-1610 | PX979372-979373 | *Cadophora* sp. | MT529236 | 100 | 4 | X06 | R |
| Asteraceae | *Hieracium umbellatum* | LYZ1335-1338 | PX979239-979242 | *Cadophora luteo-olivacea* | MT197382 | 99.50 | 4 | X07 | BS |
| Fabaceae | *Astragalus laxmannii* | LYZ1323-1326 | PX979227-979230 | *Coniothyrium* sp. | MK595515 | 94.73 | 10 | I13 | BS |
| Fabaceae | *Astragalus laxmannii* | LYZ1568-1569 | PX979333-979334 | *Alternaria alternata* | MF070740 | 100 | 21 | I13 | R; BS |
| Fabaceae | *Astragalus laxmannii* | LYZ1570-1571 | PX979335-979336 | *Fusarium tricinctum* species complex | OQ415196 | 100 | 8 | I13 | R; BS |
| Fabaceae | *Astragalus schanginianus* | LYZ1593-1594 | PX570701-570702 | *Fusarium acuminatum* | LC543657 | 100 | 27 | X07 | R; BS |
| Gentianaceae | *Gentiana macrophylla* | LYZ1339-1342 | PX979243-979246 | *Leptosphaeria* sp. | PP781252 | 100 | 4 | Q01 | R |
| Juncaceae | *Juncus bufonius* | LYZ1264-1271 | PX979176-979183 | *Pyrenophora leucospermi* | MK539989 | 99.68 | 43 | X06 | R; BS |
| Nitrariaceae | *Peganum harmala* | LYZ1275-1278 | PX979187-979190 | *Fusarium incarnatum-equiseti* species complex | ON209646 | 100 | 98 | I02 | R; BS |
| Nitrariaceae | *Peganum harmala* | LYZ1279-1282;  LYZ1623-1624 | PX979191-979194; PX979386-979387 | *Fusarium tricinctum* species complex | OR041600 | 100 | 52 | I02 | R; BS |
| Nitrariaceae | *Peganum harmala* | LYZ1536-1537 | PX979301-979302 | *Phoma conidiogena* | MH865146 | 100 | 4 | I02 | R; BS |
| Nitrariaceae | *Peganum harmala* | LYZ1621-1622 | PX979384-979385 | *Alternaria alternata* | MF070740 | 100 | 16 | I02 | R; BS |
| Plantaginaceae | *Plantago asiatica* | LYZ1351-1353 | PX979255-979257 | *Fusarium redolens* | PX468614 | 100 | 3 | Q01 | R |
| Plantaginaceae | *Plantago asiatica* | LYZ1599-1601 | PX979362-979364 | **Pleosporales** | GQ924022 | 94.95 | 6 | Q01 | R |
| Plantaginaceae | *Plantago asiatica* | LYZ1602 | PX979365 | *Cadophora luteo-olivacea* | MT022461 | 100 | 4 | Q01 | R |
| Plantaginaceae | *Plantago asiatica* | LYZ1513-1516 | PX979278-979281 | *Cadophora luteo-olivacea* | MN871427 | 99.84 | 8 | I09 | R |
| Rosaceae | *Potentilla anserina* | LYZ1611-1612 | PX979374-979375 | *Alpinaria rhododendri* | OR286723 | 99.79 | 5 | Q01 | R |
| Rosaceae | *Potentilla bifurca* | LYZ1260-1263 | PX979172-979175 | *Phlyctema* sp. | MW478680 | 100 | 4 | Q01 | R |
| Rosaceae | *Potentilla chinensis* | LYZ1608 | PX979371 | **Helotiales** | DQ350130 | 99.06 | 9 | I15 | R; BS |
| Rosaceae | *Potentilla fruticosa* | LYZ1517-1519 | PX979282-979284 | **Helotiales** | DQ249995 | 95.39 | 7 | Q01 | R |
| Rosaceae | *Potentilla pensylvanica* | LYZ1307-1310 | PX979211-979214 | *Seimatosporium pistaciae* | KP004464 | 100 | 15 | X07 | R; BS |
| Rosaceae | *Potentilla tanacetifolia* | LYZ1291-1294 | PX979203-979206 | *Fusarium oxysporum* species complex | MZ668437 | 100 | 12 | I14 | R; BS |

Note: R indicates root, BS indicates basal stem. Bold text indicates identification only to the intended target taxon.

Supplementary Table 2. Functional guild assignment of culturable fungi isolated from grassland plants

| Fungal taxon | Functional guild | Reference | Fungal taxon | Functional guild | Reference |
| --- | --- | --- | --- | --- | --- |
| *Albotricha* sp. | E | (Durán et al., 2021) | *Lachnum* sp. | E | (Durán et al., 2021) |
| *Alpinaria rhododendri* | S | (Karunarathna, 2020) | *Leptosphaeria* sp. | E; P | (Yamaguchi & Kataoka, 2024) |
| *Alternaria alternata* | P | (Ma et al., 2026) | *Leratiomyces laetissimus* | S | (Holec & Assollant, 2011) |
| *Alternaria chlamydosporigena* | P | (Wang & Li, 2025a) | *Limonomyces* sp. | P | (Diederich et al., 2011) |
| *Alternaria longissima* | E | (He et al., 2021) | *Marasmius* sp. | S | (Chen et al., 2025) |
| *Austropleospora keteleeriae* | S | (Lu et al., 2022) | *Microdochium bolleyi* | E | (Matušinsky et al., 2022) |
| *Cadophora luteo-olivacea* | P | (Llorens et al., 2025) | *Microdochium trichocladiopsis* | S | (Hernández-Restrepo et al., 2016) |
| *Cadophora* sp. | P | (Karpova et al., 2025) | *Microscypha* sp. | S | (Guo et al., 2024) |
| *Cercophora* sp. | S | (Crous et al., 2016) | *Neoascochyta europaea* | P | (Golzar et al., 2019) |
| *Cistella* sp. | S | (Guo et al., 2024) | *Niesslia aemula* | S | (Gams et al., 2019) |
| *Colletotrichum spaethianum* | P | (Zhang & Fan, 2025) | *Ophiosphaerella* sp. | E; P; S | (Li et al., 2018; Martinez et al., 2019) |
| *Coniochaeta* sp. | S | (Wang et al., 2025) | *Paraphaeosphaeria* sp. | E | (Li et al., 2024) |
| *Coniothyrium* | P; S | (Peng et al., 2025) | *Periconia macrospinosa* | E | (Høyer et al., 2022) |
| *Crinipellis scabella* | S | (Jaworska et al., 2012) | *Phaeosphaeria pontiformis* | E | (Paulovska et al., 2017) |
| *Darksidea alpha* | E | (He et al., 2021) | *Phaeosphaeria* sp. | E; P; S | (Hongsanan et al., 2020) |
| *Darksidea* sp. | E | (Knapp et al., 2015) | *Phialophora mustea* | E | (Unni *et al.*, 2024) |
| *Fusarium acuminatum* | P | (Shrestha et al., 2021) | *Phlyctema* sp. | E; P | (Aguilar et al., 2019) |
| *Fusarium concolor* | P; S | (Rana & Singh, 2023) | *Phoma conidiogena* | S | (Swift, 1932) |
| *Fusarium incarnatum-equiseti* species complex | P | (Avila et al., 2019) | *Plenodomus tracheiphilus* | P | (Mosca et al., 2024) |
| *Fusarium nygamai* | P | (Özer et al., 2023) | *Poaceascoma* sp. | S | (Luo et al., 2016) |
| *Fusarium oxysporum* species complex | P | (Chang et al., 2026) | *Preussia terricola* | E | (He et al., 2021) |
| *Fusarium redolens* | P | (Shrestha et al., 2021) | *Pyrenophora leucospermi* | P | (Crous et al., 2011) |
| *Fusarium tricinctum* species complex | P | (Laraba et al., 2022) | *Rhypophila decipiens* | E; P; S | (Wang & Li, 2025b) |
| *Gaeumannomyces hyphopodioides* | E | (Osborne et al., 2018) | *Seimatosporium pistaciae* | P; S | (Crous et al., 2014; Mohammadian et al., 2017) |
| *Gaeumannomyces* sp. | E; P; S | (Hill et al., 2025) | *Septoriella* sp. | S | (Wang et al., 2018) |
| *Heteroconium* sp. | E; S | (Lahlali et al., 2014) | *Slopeiomyces cylindrosporus* | E | (Høyer et al., 2022) |
| *Knufia tsunedae* | S | (Crous et al., 2013) |  |  |  |

Note: E means endophytes, S means saprobes, P means pathogens.

Supplementary Table 3. Significant fungus-host enriched pairs based on adjusted residual analyses (adjusted residual ≥ |2.0|) (68 significant pairs)

| Fungal species | Host plant | Adjusted residual | Fungal species | Host plant | Adjusted residual |
| --- | --- | --- | --- | --- | --- |
| *Albotricha* sp. | *Leymus chinensis* | 4.5 | *Heteroconium* sp. | *Carex turkestanica* | 5.3 |
| *Alpinaria rhododendri* | *Potentilla anserina* | 9.2 | *Knufia tsunedae* | *Achnatherum splendens* | 2.9 |
| *Alternaria alternata* | *Astragalus laxmannii* | 3.6 | *Lachnum* sp. | *Leymus chinensis* | 4.5 |
| *Alternaria alternata* | *Peganum harmala* | 3.1 | *Leptosphaeria* sp. | *Elymus nutans* | 3.1 |
| *Alternaria chlamydosporigena* | *Poa albertii* | 9.2 | *Leptosphaeria* sp. | *Gentiana macrophylla* | 6.5 |
| *Alternaria longissima* | *Agropyron cristatum* | 3.7 | *Leratiomyces laetissimus* | *Agropyron cristatum* | 2.4 |
| *Austropleospora keteleeriae* | *Artemisia japonica* | 6.5 | *Leratiomyces laetissimus* | *Leymus secalinus* | 4.5 |
| *Cadophora luteo-olivacea* | *Hieracium umbellatum* | 5.3 | *Limonomyces* sp. | *Stipa purpurea* | 6.5 |
| *Cadophora luteo-olivacea* | *Plantago asiatica* | 5.2 | *Marasmius* sp. | *Agropyron cristatum* | 3.7 |
| *Cadophora* sp. | *Artemisia japonica* | 6.5 | *Microdochium bolleyi* | *Achnatherum splendens* | 2.9 |
| *Cercophora* sp. | *Elymus nutans* | 4.5 | *Microdochium trichocladiopsis* | *Leymus chinensis* | 4.5 |
| *Cistella* sp. | *Carex kokanica* | 6.5 | *Microscypha* sp. | *Carex haematostoma* | 6.5 |
| *Colletotrichum spaethianum* | *Stipa penicillata* | 5.3 | *Neoascochyta europaea* | *Elymus nutans* | 4.5 |
| *Coniochaeta* sp. | *Carex alatauensis* | 6.5 | *Niesslia aemula* | *Leymus flexus* | 6.5 |
| *Coniothyrium* sp. | *Astragalus laxmannii* | 5.3 | *Ophiosphaerella* sp. | *Achnatherum psilantherum* | 2.7 |
| *Crinipellis scabella* | *Achnatherum splendens* | 2.9 | *Ophiosphaerella* sp. | *Leymus flexus* | 6.5 |
| *Darksidea alpha* | *Achnatherum splendens* | 2.6 | *Paraphaeosphaeria* sp. | *Carex turkestanica* | 5.3 |
| *Darksidea alpha* | *Elymus nutans* | 2.0 | *Periconia macrospinosa* | *Agropyron cristatum* | 3.7 |
| *Darksidea* sp. | *Carex kokanica* | 3.1 | *Phaeosphaeria pontiformis* | *Agropyron cristatum* | 3.7 |
| *Fusarium acuminatum* | *Astragalus schanginianus* | 9.2 | *Phaeosphaeria pontiformis* | *Bromus inermis* | 2.7 |
| *Fusarium concolor* | *Achnatherum regelianum* | 4.0 | *Phaeosphaeria pontiformis* | *Leymus chinensis* | 3.1 |
| *Fusarium incarnatum-equiseti* species complex | *Achnatherum psilantherum* | 2.1 | *Phialophora mustea* | *Stipa penicillata* | 5.3 |
| *Fusarium incarnatum-equiseti* species complex | *Achnatherum regelianum* | 2.1 | *Phlyctema* sp. | *Potentilla bifurca* | 9.2 |
| *Fusarium incarnatum-equiseti* species complex | *Peganum harmala* | 2.4 | *Phoma conidiogena* | *Peganum harmala* | 4.5 |
| *Fusarium nygamai* | *Achnatherum regelianum* | 4.0 | *Plenodomus tracheiphilus* | *Achillea millefolium* | 9.2 |
| *Fusarium oxysporum* species complex | *Leymus secalinus* | 4.5 | *Poaceascoma* sp. | *Achnatherum regelianum* | 2.7 |
| *Fusarium oxysporum* species complex | *Potentilla tanacetifolia* | 6.5 | *Preussia terricola* | *Achnatherum psilantherum* | 2.7 |
| *Fusarium redolens* | *Plantago asiatica* | 4.5 | *Preussia terricola* | *Achnatherum regelianum* | 2.7 |
| *Fusarium tricinctum* species complex | *Achnatherum psilantherum* | 2.1 | *Preussia terricola* | *Leymus flexus* | 3.6 |
| *Fusarium tricinctum* species complex | *Astragalus laxmannii* | 2.8 | *Pyrenophora leucospermi* | *Juncus bufonius* | 9.2 |
| *Fusarium tricinctum* species complex | *Peganum harmala* | 2.4 | *Rhypophila decipiens* | *Deyeuxia pyramidalis* | 9.2 |
| *Gaeumannomyces hyphopodioides* | *Agropyron cristatum* | 3.7 | *Seimatosporium pistaciae* | *Potentilla pensylvanica* | 9.2 |
| *Gaeumannomyces* sp. | *Carex turkestanica* | 5.3 | *Slopeiomyces cylindrosporus* | *Achnatherum psilantherum* | 2.7 |
| *Heteroconium* sp. | *Carex turkestanica* | 5.3 | *Slopeiomyces cylindrosporus* | *Bromus inermis* | 2.7 |

**References**

Aguilar, C. G., Mazzola, M., and Xiao, C. L. (2019). Timing of perennial canker development in apple trees caused by Neofabraea perennans and Neofabraea kienholzii. *Plant Dis.* 103, 555–562. doi: 10.1094/PDIS-06-18-0935-RE

Avila, C. F., Moreira, G. M., Nicolli, C. P., Gomes, L. B., Abreu, L. M., Pfenning, L. H., et al. (2019). Fusarium incarnatum-equiseti species complex associated with Brazilian rice: phylogeny, morphology and toxigenic potential. *Int. J. Food Microbiol*. 306, 108267. doi: 10.1016/j.ijfoodmicro.2019.208627

Chang, A., Chang, C. W., Wu, C. C., Lin, K. H., Chookoh, N., Unartngam, J., et al. (2026). Orchid diseases caused by Fusarium oxysporum species complex in Taiwan. *Front. Plant Sci*. 16, 1630094. doi: 10.3389/fpls.2025.1630094

Chen, H., Xu, Y. Q., Zeng, H., Hu, Y. P., Wang, S. N., and Yan, J. Q. (2025). Four new species of Marasmius subgenus Globulares (Marasmiaceae, Agaricales) from subtropical regions of China. *MycoKeys* 120, 317–338. doi: 10.3897/mycokeys.120.157997

Crous, P. W., Summerell, B. A., Swart, L., Denman, S., Taylor, J. E., Bezuidenhout, C. M., et al. (2011). Fungal pathogens of Proteaceae. *Persoonia* 27, 20–45. doi: 10.3767/003158511X606239

Crous, P. W., Wingfield, M. J., Burgess, T. I., Hardy, G. E., Crane, C., Barrett, S., et al. (2016). Fungal planet description sheets: 469–557. *Persoonia* 37, 218–403. doi: 10.3767/003158516X694499

Crous, P. W., Wingfield, M. J., Guarro, J., Cheewangkoon, R., van der Bank, M., Swart, W. J., et al. (2013). Fungal planet description sheets: 154–213. *Persoonia* 31, 188–296. doi: 10.3767/003158513X675925

Crous, P. W., Wingfield, M. J., Schumacher, R. K., Summerell, B. A., Giraldo, A., Gené, J., et al. (2014). Fungal planet description sheets: 281–319. *Persoonia* 33, 212–289. doi: 10.3767/003158514X685680.

Diederich, P., Lawrey, J. D., Sikaroodi, M., and Gillevet, P. M. (2011). A new lichenicolous teleomorph is related to plant pathogens in Laetisaria and Limonomyces (Basidiomycota, Corticiales). *Mycologia* 103, 525–533. doi: 10.3852/10-255

Durán, M., San, E. L., and Canals, R. M. (2021). Comparison of culturing and metabarcoding methods to describe the fungal endophytic assemblage of Brachypodium rupestre growing in a range of anthropized disturbance regimes. *Biol. (Basel)* 10, 1246. doi: 10.3390/biology10121246

Gams, W., Stielow, B., Gräfenhan, T., and Schroers, H. J. (2019). The ascomycete genus Niesslia and associated monocillium-like anamorphs. *Mycol. Prog*. 18, 5–76. doi: 10.1007/s11557-018-1459-5

Golzar, H., Thomas, G., Jayasena, K. W., Wright, D., Wang, C., and Kehoe, M. (2019). Neoascochyta species cause leaf scorch on wheat in Australia. *Australas. Plant Dis*. 14, 1. doi: 10.1007/s13314-018-0332-3

Guo, J., Xie, Z., Meng, Q., Xu, H., Peng, Q., Wang, B., et al. (2024). Distribution of rhizosphere fungi of Kobresia humilis on the Qinghai-Tibet Plateau. *PeerJ* 12, e16620. doi: 10.7717/peerj.16620

He, C., Wang, W., Hou, J., and Li, X. (2021). Dark septate endophytes isolated from wild licorice roots frown in the desert regions of northwest China enhance the growth of host plants under water deficit stress. *Front. Microbiol*. 12, 522449. doi: 10.3389/fmicb.2021.522449

Hernández-Restrepo, M., Groenewald, J. Z., and Crous, P. W. (2016). Taxonomic and phylogenetic re-evaluation of Microdochium, Monographella and Idriella. *Persoonia* 36, 57–82. doi: 10.3767/003158516X688676

Hill, R., Smith, D., Canning, G., Grey, M., Hammond-Kosack, K. E., and McMullan, M. (2025). Starship giant transposable elements cluster by host taxonomy using k-mer-based phylogenetics. *G3 (Bethesda)* 15, jkaf082. doi: 10.1093/g3journal/jkaf082

Holec, J., and Assollant, J. (2011). *Fungi europaei* (Origgio: Candusso Edizioni).

Hongsanan, S., Hyde, K. D., Phookamsak, R., Wanasinghe, D. N., McKenzie, E. H. C., Sarma, V. V., et al. (2020). Refined families of Dothideomycetes: orders and families incertae sets in Dothideomycetidae. *Fungal Divers*. 105, 17–318. doi: 10.1007/s13225-020-00462-6

Høyer, A. K., Jørgensen, H. J. L., Hodkinson, T. R., and Jensen, B. (2022). Fungal endophytes isolated from Elymus repens, a wild relative of barley, have potential for biological control of Fusarium culmorum and Pyrenophora teres in barley. *Pathogens* 11, 1097. doi: 10.3390/pathogens11101097

Jaworska, J., Luszczynski, J., Luszczynska, B., and Tomaszewska, A. (2012). Macromycetes indicator species for xerothermic grasslands of the Checiny district. *Acta Agrobot* 65, 63–70. doi: 10.5586/aa.2012.044

Karpova, D., Belkina, D., Porotikova, E., Yurchenko, E., and Vinogradova, S. (2025). Metagenomic study of the grapevine decline detected a cocktail of fungi associated with grapevine trunk diseases. *Plants (Basel)* 14, 3722. doi: 10.3390/plants14243722

Karunarathna, S. C. (2020). Addition to Melanommataceae: a new geographical record of Alpinaria rhododendri from Shangri La, China. *AJOM* 3, 335–344. doi: 10.5943/ajom/3/1/8

Knapp, D. G., Kovács, G. M., Zajta, E., Groenewald, J. Z., and Crous, P. W. (2015). Dark septate endophytic pleosporalean genera from semiarid areas. *Persoonia* 35, 87–100. doi: 10.3767/003158515X687669

Lahlali, R., McGregor, L., Song, T., Gossen, B. D., Narisawa, K., and Peng, G. (2014). Heteroconium chaetospira induces resistance to clubroot via upregulation of host genes involved in jasmonic acid, ethylene, and auxin biosynthesis. *PLoS One* 9, e94144. doi: 10.1371/journal.pone.0094144

Laraba, I., Busman, M., Geiser, D. M., and O'Donnell, K. (2022). Phylogenetic diversity and mycotoxin potential of emergent phytopathogens within the Fusarium tricinctum species complex. *Phytopathology* 112, 1284–1298. doi: 10.1094/PHYTO-09-21-0394-R

Li, Q., Yang, P. Y., Peng, C., Zhang, X. J., Jiang, Y. T., Li, Y. P., et al. (2024). New meroterpenoids and polyketides from the endophytic fungus Paraphaeosphaeria sp. C-XB-J-1 and their anti-inflammatory and SARS-CoV-2 Mpro inhibitory activities. *Bioorg. Chem*. 147, 107315. doi: 10.1016/j.bioorg.2024.107315.

Li, Y., Zhu, R., Zhang, J., Xie, F., Wang, X., Xu, K., et al. (2018). Ophiosphaerellins A-I, Polyketide-derived compounds from the endolichenic fungus Ophiosphaerella korrae. *ACS Omega* 3, 176–180. doi: 10.1021/acsomega.7b01668

Llorens, E., López-Moral, A., Antón-Domínguez, B. I., Jiménez-Urbano, J. P., Trapero, A., and Agustí-Brisach, C. (2025). Interaction among Cadophora luteo-olivacea, Phaeoacremonium minimum, and Pseudophaeomoniella oleicola in olive multi-infections and cultivar susceptibility. *Plant Dis*. 109, 2147–2153. doi: 10.1094/PDIS-11-24-2466-RE

Lu, L., Karunarathna, S. C., Dai, D. Q., Xiong, Y. R., Suwannarach, N., Stephenson, S. L., et al. (2022). Description of four novel species in Pleosporales associated with coffee in Yunnan, China. *J. Fungi (Basel)* 8, 1113. doi: 10.3390/jof8101113

Luo, Z. L., Bahkali, A. H., Liu, X. Y., Phookamsak, R., Zhao, Y. C., Zhou, D. Q., et al. (2016). Poaceascoma aquaticum sp. nov. (Lentitheciaceae), a new species from submerged bamboo in freshwater. *Phytotaxa* 253, 71–80. doi: 10.11646/phytotaxa.253.1.5

Ma, C., Han, K., and Yan, D. (2026). The whole-genome sequence and analysis of Alternaria alternata strain LG038 isolated from Capsicum annuum. *Microbiol. Resour. Announc* 4, e0119525. doi: 10.1128/mra.01195-25

Martinez, I. J. F., Flores, F. J., Koch, A. R., Garzón, C. D., and Walker, N. R. (2019). Multiplex end-point PCR for the detection of three species of Ophiosphaerella causing spring dead spot of bermudagrass. *Plant Dis*. 103, 2010–2014. doi: 10.1094/PDIS-10-18-1727-RE

Matušinsky, P., Sedláková, B., and Bleša, D. (2022). Compatible interaction of Brachypodium distachyon and endophytic fungus Microdochium bolleyi. *PLoS One* 17, e0265357. doi: 10.1371/journal.pone.0265357

Mohammadian, E., Babai Ahari, A., Arzanlou, M., Oustan, S., and Khazaei, S. H. (2017). Tolerance to heavy metals in filamentous fungi isolated from contaminated mining soils in the Zanjan Province, Iran. *Chemosphere* 185, 290–296. doi: 10.1016/j.chemosphere.2017.07.022

Mosca, A., Dimaria, G., Nicotra, D., Modica, F., Massimino, M. E., Catara, A. F., et al. (2024). Soil microbial communities in lemon orchards affected by citrus mal secco disease. *Genes (Basel)* 15, 824. doi: 10.3390/genes15070824

Osborne, S. J., McMillan, V. E., White, R., and Hammond-Kosack, K. E. (2018). Elite UK winter wheat cultivars differ in their ability to support the colonization of beneficial root-infecting fungi. *J. Exp. Bot*. 69, 3103–3115. doi: 10.1093/jxb/ery136

Özer, G., Erper, İ., Yıldız, Ş., Bozoğlu, T., Zholdoshbekova, S., Alkan, M., et al. (2023). Fungal pathogens associated with crown and root rot in wheat-growing areas of northern Kyrgyzstan. *J. Fungi (Basel)* 9, 124. doi: 10.3390/jof9010124

Paulovska, L., Bankina, B., Roga, A., and Fridmanis, D. (2017). “*The incidence of wheat crown rot depending on agronomic practices*,” in Research for Rural Development – Proceedings of the Annual 23rd International Scientific Conference (Jelgava).

Peng, C., Li, Y., Yu, H., He, H., Cheng, Y., Sun, S., et al. (2025). Synergistic recruitment of symbiotic fungi by potting and Scleroderma bovista inoculation suppresses pathogens in hazel rhizosphere microbiomes. *Microorganisms* 13, 1063. doi: 10.3390/microorganisms13051063

Rana, S., and Singh, S. K. (2023). Insights into the genomic architecture of a newly discovered endophytic Fusarium species belonging to the Fusarium concolor complex from India. *Front. Microbiol*. 14, 1266620. doi: 10.3389/fmicb.2023.1266620

Shrestha, S., Poudel, R. S., and Zhong, S. (2021). Identification of fungal species associated with crown and root rots of wheat and evaluation of plant reactions to the pathogens in north Dakota. *Plant Dis*. 105, 3564–3572. doi: 10.1094/PDIS-11-20-2412-RE

Swift, M. E. (1932). Phoma conidiogena on box. *Mycologia* 24, 199–206. doi: 10.1080/00275514.1932.12020610

Unni, S., Kommu, P., Aouti, S., Nalli, Y., Bharath, M. S., Ali, A., et al. (2024). Structural insights into the modulation of SOD1 aggregation by a fungal metabolite Phialomustin-B: Therapeutic potential in ALS. *PLoS One* 19, e0298196. doi: 10.1371/journal.pone.0298196

Wang, H., Kuang, S., Lang, Q., and Yu, W. (2018). Effects of aged oil sludge on soil physicochemical properties and fungal diversity revealed by high-throughput sequencing analysis. *Archaea* 2018, 9264259. doi: 10.1155/2018/9264259

Wang, T. T., and Li, Y. Z. (2025a). First report of Alternaria chlamydosporigena causing Poa poophagorum root rot in China. *Plant Dis*. 110, 236. doi: 10.1094/PDIS-07-25-1538-PDN

Wang, T. T., and Li, Y. Z. (2025b). First report of Rhypophila decipiens causing Deyeuxia pyramidalis root rot in Qinghai, China. *Plant Dis*. 110, 233. doi: 10.1094/pdis-08-25-1609-pdn

Wang, Y., Ta, N., Zhang, H., Li, M., Liu, S., Gong, J., et al. (2025). Beyond one-size-fits-all approach: How do various harvesting strategies shape soil microbial diversity in Larix gmelinii (Daxinganling larch) forests of the Greater Khingan Mountains? *Front. Microbiol*. 16, 1654005. doi: 10.3389/fmicb.2025.1654005

Yamaguchi, T., and Kataoka, R. (2024). Novel endophytic fungus Leptosphaeria sp. strain T-2 improves plant growth and environmental stress tolerance. *Stress Biol*. 4, 52. doi: 10.1007/s44154-024-00186-6

Zhang, W., and Fan, X. (2025). Diversity and distribution of Colletotrichum species causing anthracnose in China. *J. Fungi (Basel)* 11, 781. doi: 10.3390/jof11110781
